# Brown adipose activation and reversible beige coloration in adipose tissue with multiple accumulations of ^18^F‐fluorodeoxyglucose in sporadic paraganglioma: A case report

**DOI:** 10.1002/ccr3.2259

**Published:** 2019-06-11

**Authors:** Eriko Terada, Kenji Ashida, Kenji Ohe, Shohei Sakamoto, Nao Hasuzawa, Masatoshi Nomura

**Affiliations:** ^1^ Department of Medicine and Bioregulatory Science, Graduate School of Medical Sciences Kyushu University Fukuoka Japan; ^2^ Division of Endocrinology and Metabolism, Department of Internal Medicine Kurume University School of Medicine Kurume, Fukuoka Japan; ^3^ Faculty of Pharmaceutical Sciences Fukuoka University Fukuoka Japan

**Keywords:** beige adipose tissue, catecholamine, fluorodeoxyglucose, paraganglioma, pheochromocytoma

## Abstract

In pheochromocytoma/paraganglioma, nontumorous high ^18^F‐fluorodeoxyglucose accumulations are observed in both beige and brown adipose tissues. Recognizing this feature of ^18^F‐fluorodeoxyglucose accumulation can help physicians make precise diagnoses and help them avoid the pitfalls of a false‐positive ^18^F‐fluorodeoxyglucose positron emission tomography result, preventing unnecessary interventions.

## 1 BACKGROUND

Pheochromocytomas/paragangliomas (PPGLs) are endocrine tumors derived from tumorigenic chromaffin cells of the adrenal medulla or extraadrenal ganglia. Most PPGLs secrete catecholamines and represent high circulating catecholamine levels, leading to overstimulation of adrenergic receptors and high mortality due to hypertension, stroke, and cardiomyopathy‐related congestive heart disease.^1^

Although most PPGLs are sporadic, more than one‐third of PPGL cases have been considered to have genetic backgrounds, such as von Hippel‐Lindau disease, neurofibromatosis type 1, multiple endocrine neoplasia type 2, mutations in succinate dehydrogenase complex subunit genes, subunit cofactor, transmembrane protein 127, and myc‐associated factor X.^1^, ^2^ As one‐fourth of PPGLs have been reported to be malignant,^3^ ^18^F‐fluorodeoxyglucose (FDG) positron emission tomography (PET) examination has been recommended for detecting metastatic lesions, to ensure precise interventions.^4^ FDG‐PET could be used to identify multiple affected lesions in these cases.^1^, ^2^

High FDG accumulation has been reported in activated brown or beige‐colored white adipose tissue caused by catecholamines^5^, ^6^, ^7^ or cold exposure.^8^ Successful resection of PPGLs, resulting in amelioration of hyper‐catecholamine levels, leads to disappearance of high FDG accumulations in adipose tissues. However, beige coloration of human adipose tissue is still a controversial issue. Therefore, in this PPGL case, we examined adipose tissue that had changed from white to beige using immunohistochemical staining.

## 2 CASE PRESENTATION

**Figure 1:**
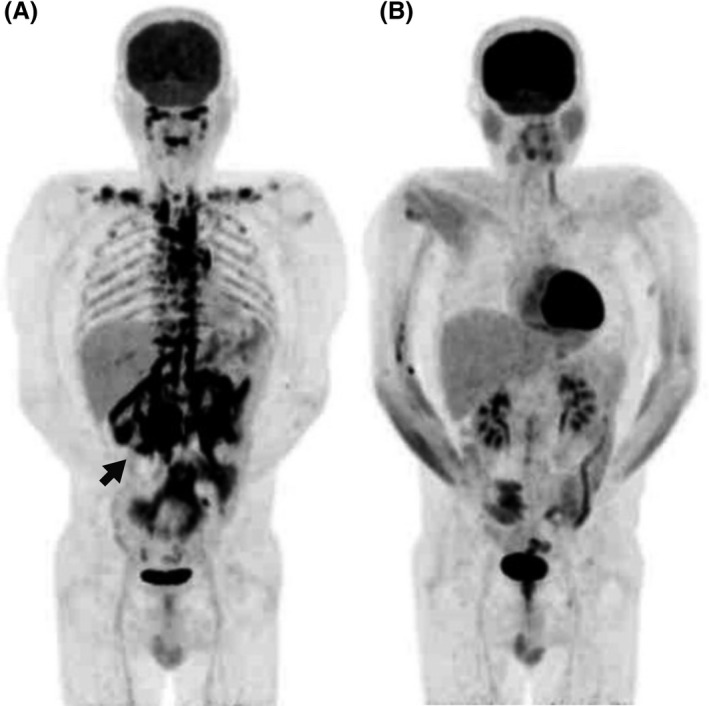
Planar view of ^18^F‐fluorodeoxyglucose (FDG) positron emission tomography. A, Multiple high accumulations of FDG not only in the retroperitoneal tumor (arrow) but also in the peritracheal, upper mediastinal, supraclavicular, perispinal, periaortic, and perirenal spaces; B, disappearance of multiple FDG accumulations 1 mo after the resection of the right retroperitoneal paraganglioma

**Figure 2:**
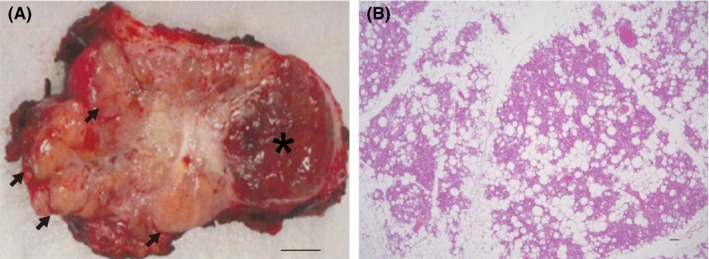
Paraganglioma and beige‐colored peritumoral fat tissues. A, The resected retroperitoneal tumor (*) showing white peritumoral fat tissues that have changed to beige tissues (arrows); scale bar represents 10 mm; B, hematoxylin‐eosin staining of peritumoral fat tissues showing enlarged islets of multilocular fat cells within white fat depots; scale bar represents 100 μm

**Figure 3:**
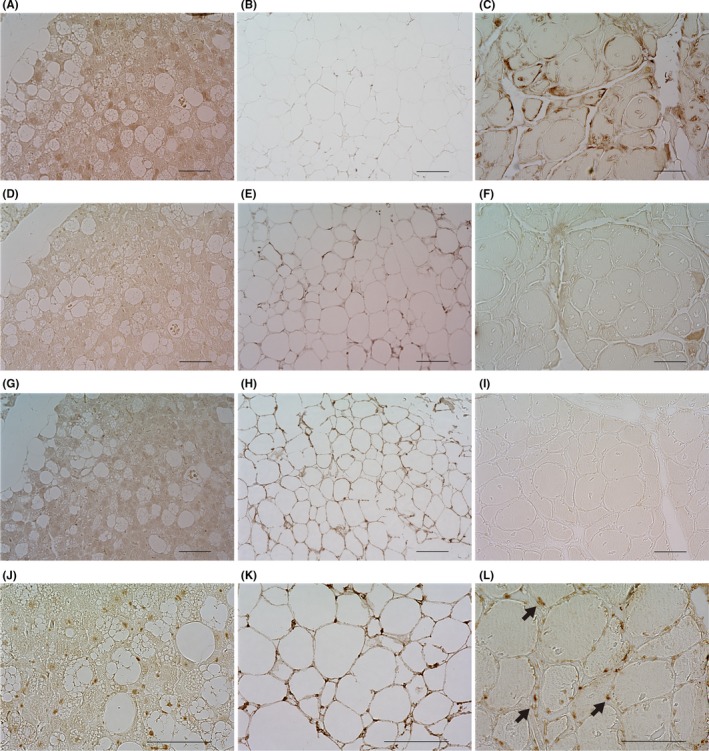
Immunohistochemical staining of beige‐colored peritoneal fat tissues. Each sample of peritumoral fat tissue (A, D, G, J), human white adipose tissue (B, E, H, K), and human skeletal muscle (C, F, I, L) is stained using the following specific anti‐human antibodies: (A‐C) UCP1, (D‐F) PGC1α, (G‐I) CITED1, and (J‐L) MYF5. White adipose tissues, attached to the resected para‐aortic paraganglioma, show positive staining for UCP1 (A), PGC1α (D), CITED1 (G), and MYF5 (arrows) (J). White adipose tissues show positive staining for PGC1a (E) and CITED1 (H). Skeletal muscle tissues show positive staining for UCP1 (C) and MYF5 (arrows) (L). Scale bars represent 100 μm. Samples of human white adipose tissues and skeletal muscles were obtained from BioChain Institute Inc, CA. CITED1, CBP/p300‐interacting transactivator with Glu/Asp‐rich carboxy‐terminal domain 1; MYF5, myogenic factor 5; PGC1α, peroxisome proliferator‐activated receptor‐γ coactivator 1‐α; UCP1, uncoupling protein 1

A 35‐year‐old man was admitted to a tertiary care center with right upper abdominal pain and palpitations. He had been diagnosed with hypertension and diabetes mellitus 8 years earlier. Computed tomography revealed a right retroperitoneal tumor measuring 7 cm in diameter. Urinary noradrenaline and normetanephrine levels were high (1.38 [reference range, 0.048‐0.168] and 2.13 [reference range, 0.09‐0.33] mg/d, respectively).^9^ ^123^I‐metaiodobenzylguanidine (MIBG) scintigraphy showed no accumulation in the tumor, although magnetic resonance imaging showed elevated T2‐signal intensity. FDG‐PET showed high accumulation in the right retroperitoneal tumor with multiple accumulations in the retroperitoneal, peritracheal, upper mediastinal, and perispinal spaces bilaterally (Figure 1A), whereas ^111^In‐octreotide only accumulated within the tumor. The patient had neither familial history of hereditary PPGL nor any significant genetic mutations in the genes encoding succinate dehydrogenase complex subunits B and D.^9^ He was diagnosed with right retroperitoneal paraganglioma and underwent surgery for removal of the retroperitoneal tumor. Histopathological examination showed a paraganglioma and beige‐colored peritumoral fat tissues (Figure 2). On immunohistochemical staining, the beige‐colored peritoneal fat tissues were positive for uncoupling protein 1, peroxisome proliferator‐activated receptor‐γ coactivator 1‐α, CBP/p300‐interacting transactivator with Glu/Asp‐rich carboxy‐terminal domain 1, and myogenic factor 5 (Myf5) (Figure 3).

Catecholamine levels immediately returned to normal within a month, and the FDG accumulations disappeared 1 month after surgery (Figure 1B). Postoperatively, he stopped experiencing palpitations and right abdominal pain, and the administration of doxazosin, which was started after the PPGL diagnosis, was discontinued. To date, the patient has remained symptom‐free and recurrence‐free for 3 years.

## 3 DISCUSSION

Multiple accumulations of ^18^F‐fluorodeoxyglucose were observed in a patient with pheochromocytoma/paraganglioma. High circulating catecholamine levels likely caused brown adipose activation and beige coloration of white adipose tissue, which disappeared postoperatively. Although hereditary and malignant pheochromocytoma/paraganglioma should not be overlooked, physicians should also be aware of the potential for misdiagnosis.

Increased FDG uptake, reflecting the activation of brown adipose tissues and beige coloration of white adipose tissues,^8^, ^10^ disappeared after the right peritoneal paraganglioma was resected. β3‐adrenergic stimulation of adipocytes is known to increase glucose uptake both dependently and independently of mitochondrial activation,^11^ manifesting as high FDG accumulation.^7^ The present case demonstrated the potential of white adipose tissues to develop into beige adipose tissue by mitochondrial activation following β3‐adrenergic receptor stimulation^8^, ^12^, ^13^ in PPGL. When multiple accumulations of FDG are observed, physicians should be careful to avoid misdiagnosing false‐positive lesions as malignant metastases^14^ or multiple PPGL lesions.^15^ Therefore, if multiple FDG accumulations do not represent malignant or multiple tumors, physicians should try to avoid interventions such as chemotherapy for malignant PPGLs, exploration and resection of the FDG‐accumulating mass in the body, or radiation therapy with the ^131^I‐MIBG isotope.

Pathological examination revealed beige coloration of adipocytes in the peritumoral adipose tissue (Figure 2A,B).^16^ Although brown adipose cells and beige adipose cells have been reported to be derived from Myf5‐positive myogenic cells and Myf5‐negative white adipose cells, respectively,^6^, ^8^ we found that the human beige cells detected by FDG accumulation were derived from Myf5‐lineage cells^16^ (Figure 3A‐L). These results may help refine the currently controversial definition of beige cells (Figure 3A,D,G,J),^12^, ^17^, ^18^ and beige adipose tissue may be consistent with heterogeneous adipose cells, which show various metabolism‐related gene expressions.

In conclusion, PPGL can show multiple strong, nontumorous FDG accumulations in brown and beige adipose tissues due to β3‐adrenergic receptor stimulation. Examinations tailored to each condition and careful interpretation of results are needed for proper diagnosis,^10^ although anatomical and functional imaging modalities based on genetic information are now recommended.^9^ In addition, annual follow‐ups for at least 10 years are now recommended for paraganglioma, owing to its potential for malignancy.^19^

## CONFLICT OF INTEREST

None declared.

## AUTHOR CONTRIBUTIONS

ET: the physiotherapist in charge of the patient, collected the data and revised the manuscript. KA: drafted the manuscript and revised the manuscript. ET and KA: contributed equally to the manuscript. KO: reviewed and revised the manuscript. SS: reviewed the manuscript, interpreted the immunohistochemical imaging, and gave various suggestions. NH: analyzed the adipose tissue with immunohistochemical staining and revised the manuscript. MN: contributed to drafting and revising the manuscript.

## ETHICAL APPROVAL

All the procedures performed in this study were in accordance with the ethical standards of the institutional review board of the Kyushu University Hospital and with the principles of the Declaration of Helsinki 2013. Approval by an ethics review board was not required for this case report.

Consent: The patient provided informed consent for publication of this case report and any accompanying images. A written consent form was obtained.
